# Actomyosin-Based Nanodevices for Sensing and Actuation: Bridging Biology and Bioengineering

**DOI:** 10.3390/bios15100672

**Published:** 2025-10-04

**Authors:** Nicolas M. Brunet, Peng Xiong, Prescott Bryant Chase

**Affiliations:** 1 Department of Psychology, California State University, San Bernardino, CA 92407, USA; nicolas.brunet@csusb.edu; 2 Department of Physics, Florida State University, Tallahassee, FL 32306, USA; pxiong@fsu.edu; 3 Department of Biological Science, Florida State University, Tallahassee, FL 32306, USA

**Keywords:** actomyosin, biomolecular motors, nanoscale transport, bionanotechnology, biosensing, biohybrid systems

## Abstract

The actomyosin complex—nature’s dynamic engine composed of actin filaments and myosin motors—is emerging as a versatile tool for bio-integrated nanotechnology. This review explores the growing potential of actomyosin-powered systems in biosensing and actuation applications, highlighting their compatibility with physiological conditions, responsiveness to biochemical and physical cues and modular adaptability. We begin with a comparative overview of natural and synthetic nanomachines, positioning actomyosin as a uniquely scalable and biocompatible platform. We then discuss experimental advances in controlling actomyosin activity through ATP, calcium, heat, light and electric fields, as well as their integration into in vitro motility assays, soft robotics and neural interface systems. Emphasis is placed on longstanding efforts to harness actomyosin as a biosensing element—capable of converting chemical or environmental signals into measurable mechanical or electrical outputs that can be used to provide valuable clinical and basic science information such as functional consequences of disease-associated genetic variants in cardiovascular genes. We also highlight engineering challenges such as stability, spatial control and upscaling, and examine speculative future directions, including emotion-responsive nanodevices. By bridging cell biology and bioengineering, actomyosin-based systems offer promising avenues for real-time sensing, diagnostics and therapeutic feedback in next-generation biosensors.

## 1. Introduction

Over the past few decades, the idea of building machines at the scale of molecules—nanotechnology—has evolved from science fiction into a vibrant and fast-growing scientific and engineering field [1]. The use of biologically inspired and natural molecular machines is thus being explored in areas as diverse as medicine, biology, chemistry and materials science. Foundational visions of the field emphasized the potential of nanoscale machinery and self-assembly [1,2,3,4], providing a conceptual framework that continues to inspire both synthetic and biologically inspired nanodevices.

Whether naturally occurring or engineered, nanomachines are extremely small systems designed to convert energy into motion. They can be powered by chemical reactions, light, heat, or electrical inputs and are often capable of performing tasks autonomously or semi-independently. Their potential in medicine is especially exciting, offering possibilities for:

(1) Delivering drugs to precise locations in the body.
(2) Monitoring physiological signals through molecular-scale sensors
(3) Detecting diseases through tiny sensors.
(4) Stimulating or repairing parts of the nervous system.
(5) Transporting materials inside cells.
(6) Performing microsurgery.
(7) Aiding in tissue healing and regeneration.

**Figure 1:**
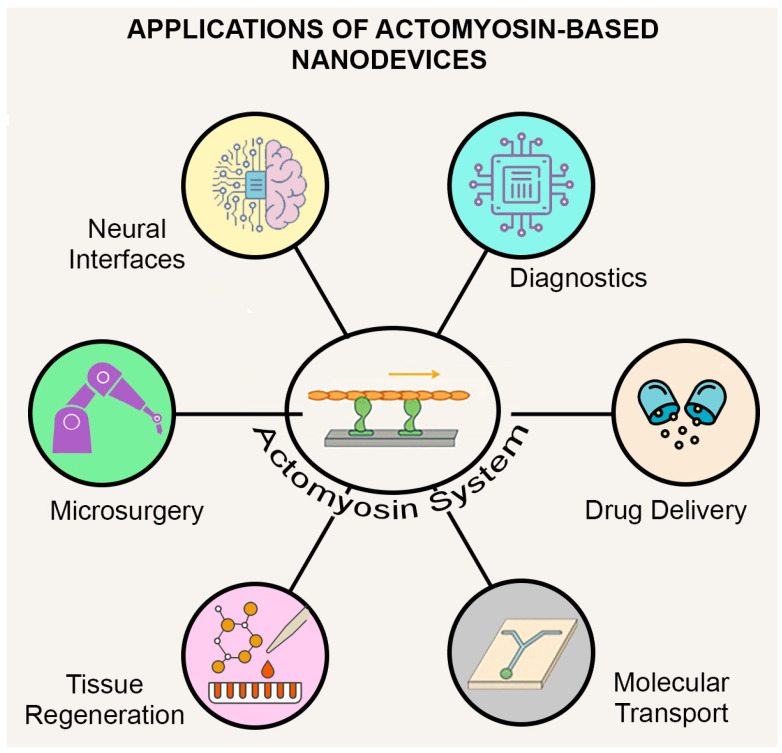
Biomedical applications of actomyosin-powered nanodevices. Biologically inspired systems (e.g., motor proteins such as myosin (green in center image) and the corresponding filaments such as actin (brown in center image) that are transported) and synthetic nanomotors are being explored for drug delivery, diagnostics, neural interfaces, microsurgery, tissue regeneration and molecular transport. Biosensing is a particularly promising near-term area, leveraging mechanical and biochemical responsiveness at the nanoscale.

These diverse opportunities are summarized in Figure 1, which highlights the broad biomedical applications where actomyosin-powered nanodevices may play a transformative role.

Of these, biosensing has emerged as a particularly promising frontier: biological systems can be used not only as targets of detection but also as active components in the sensing process itself. The fusion of mechanical responsiveness, biochemical sensitivity and nanoscale scalability opens new opportunities for biosensors that are both precise and biologically integratable [5,6,7,8].

This review begins with an overview of different types of nanomachines, including both biological and synthetic examples. It then focuses on one of biology’s most powerful and adaptable molecular motor systems: actomyosin. Found in muscle cells and across a wide range of other cell types, actomyosin combines strength, energetic efficiency, responsiveness and biocompatibility—making it especially promising for nanotechnological applications in areas including cardiovascular and neural health.

Biomolecular motors—including actomyosin—can power nanoscale transport, sorting, and sensing systems [9,10,11]. In the context of biosensing, actomyosin systems provide dynamic and reversible mechanical responses to biochemical stimuli such as ATP, calcium and pH—making them ideal candidates for developing responsive sensors that operate in physiologically relevant environments. These properties enable applications where actomyosin-powered structures can both detect and respond to biochemical cues, forming the basis of integrated sensing and actuation platforms. A recent and technically detailed review [12] focuses on actomyosin-based nanodevices, particularly in vitro motility assays and surface chemistry. The present paper complements this work by offering a broader, multidisciplinary synthesis—placing actomyosin in the wider context of biosensing, soft robotics and neuromorphic interfaces.

By bridging insights from cell biology, bioengineering and applied nanoscience, this paper highlights how actomyosin systems—powered by nature and refined by design—may serve as the basis for a new generation of smart, adaptable and biologically integrated nanodevices.

## 2. Nanotechnology at the Molecular Scale

Nanotechnology is not just one thing—it includes a wide range of tools and machines that work at incredibly small scales. Some are inspired by biology; others are designed from scratch in the lab. Table 1 provides a summary overview of the different categories of nanomachine-based devices that are being developed and the targeted applications in medicine and biology.

### 2.1. Rotary Enzymes

Some natural enzymes, such as mitochondrial ATP synthase [13,14,15,16] and bacterial flagellar motors [17,18,19,20,21], rotate as they carry out their biological functions. These naturally occurring nanomotors [17,20,21,22,23] have been adapted for use in engineered nanosystems. Enzyme-powered nanomotors (EMNMs), powered by diverse biochemical reactions, can be applied in tasks like targeted drug delivery, molecular sensing, or environmental cleanup [24]. However, the reliance of EMNMs on fuels that are not biocompatible—such as hydrogen peroxide—limits their potential for use inside the human body [25,26,27].

### 2.2. DNA Walkers

These machines use specially designed strands of DNA to “walk” along tiny tracks [8,28,29]. They are very precise and programmable, and can be used for tasks like detecting disease markers or releasing drugs at specific sites. Their main drawback is that they move slowly and need a tightly controlled environment to work properly.

### 2.3. Synthetic Molecular Motors

These are built entirely in the lab and can be triggered by light, chemicals, or other inputs [30]. They are used in “smart” materials that can change shape or function in response to their surroundings. But since they often require conditions not found inside the body, using them in living systems can be challenging [30,31,32,33,34].

### 2.4. Stimuli-Responsive Polymers

These materials undergo physical or chemical changes when exposed to specific triggers such as heat, light, or shifts in acidity. In medical contexts, they offer promise for targeted drug release or as components in artificial muscles. Despite their versatility, they still face technical challenges in achieving tissue specificity and maintaining stability in complex biological environments [35,36,37,38].

### 2.5. Nanoparticles

Particles made of gold, magnetic materials, or fluorescent compounds are used for imaging, targeted treatment and heating cancer cells [39,40,41,42,43]. Though useful, some can be toxic or hard for the body to remove.

Each of the technologies described above offer unique advantages but also come with limitations. This has motivated a growing interest in biological systems that already perform reliably within the human body—such as the actomyosin motor complex.

## 3. Molecular Motors and Their Tracks

Nature has developed effective nanoscale transport systems over millions of years. Within cells, motor proteins travel along filament networks to move cargo, assist in cell division, and maintain cellular structure with remarkable precision [44,45]. These biological mechanisms now serve as inspiration for the design of synthetic nanotechnology platforms.

### 3.1. Molecular Motor Types

Biological molecular motors belong to large, evolutionarily conserved protein families, each comprising numerous isoforms specialized for different tasks [46]. These superfamilies—myosins, kinesins and dyneins—are encoded by dozens of genes and exhibit substantial diversity in structure, function and cellular localization [46,47,48].

Three of the best-known biological motors found in eukaryotes (summarized in Table 2) are:

Myosin, which moves along actin filaments and is the molecular motor of muscle contraction and many other types of cellular movement, is particularly well suited for nanotechnology due to its biocompatibility and tunable control mechanisms [12,49,50]. In humans alone, the myosin superfamily includes over 40 genes grouped into more than 35 classes, encompassing both conventional muscle myosins (e.g., Myosin II) and unconventional variants (e.g., Myosin V, VI, X), which differ in directionality, step size, duty ratio, cargo preference and biological function [51,52,53,54] associated with localization in cells [46,47].

Kinesin, which walks toward the outer edges of cells along structures called microtubules, transports cargo over long distances, making its use feasible for applications in nanotechnology (see Khataee and Khataee [55] for its use in nanotechnology). The kinesin superfamily includes more than 45 genes in mammals and is divided into at least 14 families based on motor domain phylogeny and function [48,56]. Some kinesins move in the opposite direction (e.g., Kinesin-14), highlighting the family’s functional versatility.

Dynein, which moves in the opposite direction of most kinesins and plays roles in cell division, transporting materials and moving cilia and flagella, has also been considered for nanotech applications [55,57]. It forms large multi-subunit assemblies and is encoded by a smaller number of genes in comparison with the kinesin and myosin families. Dynein motors are exclusively minus-end directed and fall into two main types: cytoplasmic and axonemal dyneins. Both are essential for retrograde transport and motility [58,59].

Each motor protein offers a unique combination of directionality, speed and function, making them suitable building blocks for nanoscale devices requiring controlled motion. Table 2 provides a comparative overview of their key transport properties and nanotechnological relevance for the three major families of cytoskeletal motor proteins found in eukaryotes.

### 3.2. Potential Uses in Nanotechnology

Potential applications include:

(1) Transport of tiny objects, such as drug-filled, enzyme-containing, or antibody-coated vesicles, to specific locations on a surface;
(2) Sensor-triggered, localized actuation—precise responses initiated when a molecular sensor is activated;
(3) Sorting molecules in miniaturized lab-on-a-chip systems;
(4) Powering devices where these proteins carry components or move fluids.

The underlying mechanics of these systems can be reproduced in vitro using purified proteins (native or recombinantly expressed) that have been positioned on structured surfaces. By adjusting variables such as motor concentration, energy availability and the surrounding environment, it becomes possible to direct movement in a predictable and controlled fashion. Additional layers of regulations, such as light responsiveness or thermal sensitivity, can be introduced to fine-tune behavior further.

While kinesin and dynein are excellent for long-range transport, it is the actomyosin system that stands out for its balance of strength, control and compatibility with the human body. Section 4 explores this system in depth.

### 3.3. Natural vs. Synthetic Nanomotors

**Figure 2:**
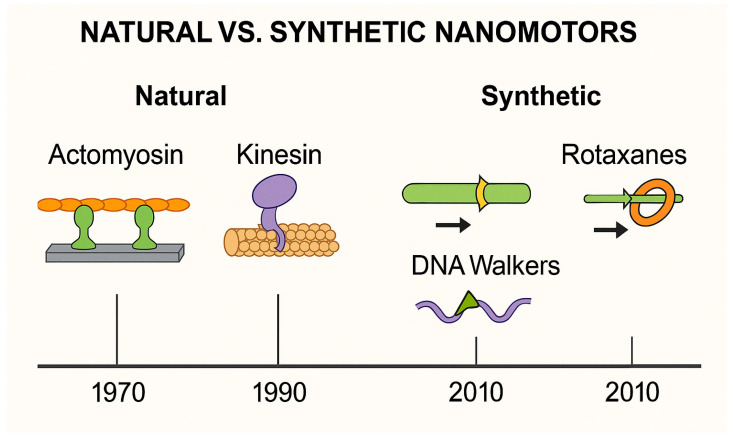
Natural vs. Synthetic Nanomotors. Comparison of key features of biological motor systems (e.g., actomyosin, kinesin and microtubules) and synthetic nanomachines (e.g., DNA walkers, rotaxanes). While synthetic systems offer programmability and modular design, biological motors provide unmatched efficiency, biocompatibility and responsiveness to physiological signals. Actomyosin stands out for its integrative potential in biomedical and hybrid nanodevice applications.

Biological motors such as myosin, kinesin and dynein convert chemical energy into mechanical work with high efficiency, responsiveness to physiological cues and excellent biocompatibility [60,61,62]. These features make them well-suited for integration into diagnostic, transport and actuation systems [11]. By contrast, synthetic nanomotors—such as DNA walkers and rotaxanes—offer design flexibility and programmability but often lack force output, speed, or operation under physiological conditions [63,64,65]. As shown in Figure 2, actomyosin systems occupy a unique niche, combining biological adaptability with engineering utility—making them ideal for hybrid nanoscale devices [66].

## 4. The Actomyosin System as a Bio-Nanomachine

### 4.1. Actomyosin’s Role in the Body

The actomyosin system is one of biology’s most reliable engines. It plays a central role in muscle contraction and also more generally in how eukaryotic cells move, divide, and maintain or change their shape. At the heart of this system are two key proteins: myosin, which acts as a molecular motor, and actin, which forms the tracks it moves along. When myosin uses energy from ATP, it pulls on actin filaments, generating motion and force at the nanoscale that can be used for cellular motion including muscle contraction, intracellular transport and modulation of cell shape including during cell division [60,67].

#### 4.1.1. Muscles: Built for Contraction

In skeletal and cardiac muscle, the actomyosin system is organized into highly regular repeating units called sarcomeres that are the reason why these muscles appear to be striated. The striations are due to thick filaments of myosin that alternate, and interdigitate with thin filaments of actin. When an electrical and/or chemical signal arrives that triggers a contraction by transiently elevating cytoplasmic Ca^2+^ concentration, these filaments slide past each other to shorten the sarcomere, producing force and movement [68,69,70]. This “sliding filament” model driven by actomyosin crossbridge cycling, proposed in the 1950s and refined over decades, remains a foundational concept in muscle physiology [71,72,73,74].

This sliding mechanism depends on a finely tuned regulatory system that controls when and how myosin interacts with actin. At the center of this regulation in eukaryotic striated muscles is tropomyosin and the troponin complex, which respond to changes in calcium ion (Ca^2+^) concentration to control access of myosin motors to binding sites on the thin filament:

(1) Molecules of tropomyosin, an α-helical coiled-coil dimeric protein, bind “head-to-tail” to form two strands on a thin filament that block myosin motors’ access to binding sites on actin at resting levels of cytoplasmic Ca^2+^ (~10^−7^ M Ca^2+^).
(2) Troponin C subunit of the troponin complex binds Ca^2+^ when a striated muscle is activated due to a transient increase in the cytoplasmic concentration of Ca^2+^ (Ca^2+^ transient).
(3) Troponin I subunit holds the troponin complex together by binding both the troponin C and troponin T subunits; the C-terminus of troponin I is located between actin and tropomyosin at resting Ca^2+^ levels, keeping tropomyosin in position to prevent formation of actomyosin crossbridges; when cytoplasmic Ca^2+^ rises during a Ca^2+^ transient, Ca^2+^ binds troponin C, which allows troponin C to bind the C-terminus of troponin I, in turn allowing tropomyosin to move on the surface of actin such that actomyosin crossbridge cycling can occur.
(4) Troponin T subunit binds the troponin complex to tropomyosin in a stoichiometry of the structural regulatory unit of 1:1:7 (troponin:tropomyosin:actin) and, due to its elongated structure, participates in communication along and between the two strands of tropomyosin on a thin filament.

Cryo-electron microscopy studies have revealed important details about how these regulatory proteins undergo structural changes in response to calcium, providing insights into their dynamic roles in contraction and relaxation [75,76,77,78,79]. When Ca^2+^ levels rise, the structure of the thin filament shifts with displacement of tropomyosin and unblocking of sites on actin that allow myosin to bind. As long as Ca^2+^ and ATP are available, the contraction continues. When Ca^2+^ declines, everything resets and the muscle relaxes [80,81].

Myosin itself exists in multiple conformational states, including an energy-conserving “super-relaxed” state that helps regulate metabolic efficiency during rest [82]. Understanding these states has implications for both basic biology and disease treatment [46].

#### 4.1.2. Beyond Muscles: A Cellular Workhorse

The same actomyosin machinery operates throughout the body in non-muscle cells, where it performs a variety of essential tasks:

(1) During cell division, actomyosin forms a contractile ring that tightens to divide one cell into two.
(2) For cell movement, it drives the extension and retraction of structures like lamellipodia and filopodia.
(3) In intracellular transport, especially in actin-rich environments such as neurons or epithelial layers, it helps to move organelles and vesicles.
(4) In mechanical sensing, it allows cells to respond to tension or pressure in their environment [83].

Different myosin isoforms specialize in these tasks, offering variations in speed, strength and cargo handling. For instance, non-muscle myosin II is critical for maintaining cell shape and enabling movement and adhesion, often working in coordination with signaling pathways to modulate cellular responses [84,85].

#### 4.1.3. Why Actomyosin Is Ideal for Nanotechnology

Actomyosin has several qualities that make it especially attractive for building nanoscale devices:

(1) It is powered by MgATP, a natural energy source, but can also utilize other nucleotides. For example, substituting deoxyATP (dATP) for ATP has been shown to enhance both the force and velocity of actomyosin interactions. This biochemical flexibility underscores the system’s adaptability and suggests opportunities to fine-tune motor output for specialized nanotechnological tasks [86,87,88].
(2) It responds to calcium, temperature and pH, offering built-in control.
(3) Although Ca^2+^ is the primary physiological regulator, other divalent cations such as Sr^2+^ and Ba^2+^ can also substitute to activate thin filaments, albeit with altered kinetics and sensitivity [89,90]. This property has been widely used experimentally and expands the biochemical flexibility of the system.
(4) It operates with remarkable efficiency, with theoretical estimates suggesting that up to 50% of chemical energy can be converted into mechanical work under optimal conditions [91].
(5) It exerts force at the piconewton level and enables motion at the nanometer scale, but organizes function across micrometer-scale cellular structures.
(6) It is reversible and durable, capable of repeating its cycle over and over.

**Figure 3:**
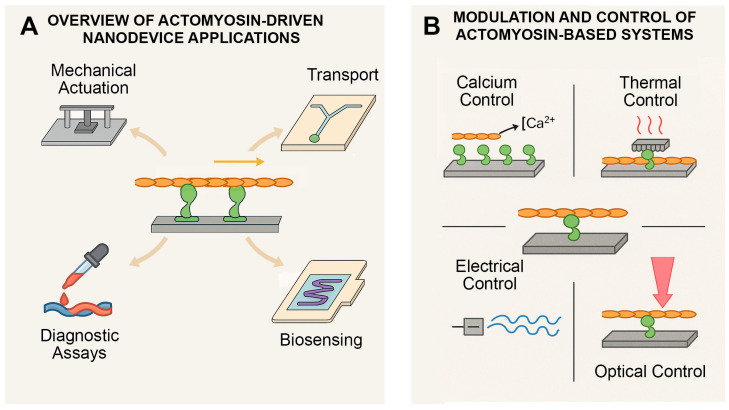
Actomyosin-Based Nanodevices and Their Modes of Control. (**A**) Overview of major applications enabled by in vitro actomyosin systems, in which actin filaments (brown filaments in both panels) glide over surface-immobilized myosin motors (green motor proteins in both panels). These biohybrid platforms support diverse functionalities—including mechanical actuation, targeted molecular transport, diagnostic assays and biosensing platforms. (**B**) External mechanisms for modulating actomyosin activity. Control inputs include biochemical cues (e.g., calcium ions, ATP), thermal triggers, electrical or electrochemical signals and optical stimulation. These strategies enable precise spatiotemporal regulation of motility, essential for responsive or programmable device integration.

These properties—summarized visually in Figure 3—position actomyosin as an ideal platform for biohybrid tools that combine biological precision with engineered versatility. Altogether, this system offers a rare combination of precision, reliability and bio-compatibility—making it a standout candidate for driving future nanotechnological tools.

### 4.2. Recreating Actomyosin Function in the Laboratory

Over the past two decades, researchers have successfully recreated the actomyosin system outside of living cells, paving the way for innovations in nanobiotechnology. These engineered setups, known collectively as in vitro motility assays, provide a controlled environment to observe and manipulate the interaction between actin filaments and myosin motors—forming the basis for biologically powered microdevices [92,93,94,95].

There are two main experimental designs commonly used:

(1) Gliding assays, where myosin motors are immobilized on a surface (typically glass or plastic), and actin filaments (typically fluorescently labeled) are allowed to glide across in the presence of ATP [96]. These setups are ideal for studying motor performance—such as speed and processivity—and for screening the effects of drugs or genetic mutations, especially in cardiac or skeletal muscle proteins.
(2) Bead assays, in which actin filaments are tethered in place while myosin-coated beads are introduced to mimic cargo transport [95,97]. These designs help to measure force generation and enable the development of systems for directional transport and targeted delivery.

As reviewed extensively by Månsson [12], myosin-based gliding assays have been optimized for surface interaction and filament stability. This review focuses on how these systems are now evolving beyond fundamental research. As illustrated in Figure 3A, these core assay formats serve as modular starting points for applications in actuation, biosensing, drug delivery and molecular transport [24]. The growing availability of purified protein components, combined with microfabrication techniques, has enabled the integration of actomyosin assays into larger platforms for medical diagnostics, drug delivery and responsive soft materials. In practice, these systems are often assembled from a mix of native and recombinant proteins: actin and myosin are typically purified from skeletal or cardiac tissue to ensure functional integrity [93,98,99], whereas regulatory proteins such as tropomyosin and troponin are more readily produced recombinantly and combined with actin filaments to create regulated, Ca^2+^-sensitive thin filaments [100,101,102]. Myosin fragments with functional motor domains (e.g., heavy meromyosin or subfragment-1) can be generated by proteolysis of purified, native myosins or, in some instances, recombinantly to facilitate surface attachment and controlled activity [93,103,104,105,106,107,108,109,110,111,112,113]. Using such combinations, reconstituted actomyosin systems have been embedded into microfluidic devices that direct cargo to specific sites or used as testbeds to study how mutations in contractile proteins alter motor function.

Importantly, these biohybrid designs not only demonstrate reliable, repeatable motion at the microscale but also offer tunable parameters—such as ATP concentration or environmental temperature—that can be customized for different use cases. Figure 3B highlights these diverse control modalities, including calcium, temperature and optical triggers—all of which contribute to programmable actomyosin performance. In summary, in vitro actomyosin assays offer more than just a window into cellular mechanics; they are increasingly serving as modular building blocks in nanotechnology. Their simplicity, versatility and compatibility with biological and synthetic components make them a powerful tool for future device development.

### 4.3. Controlling Actomyosin in Artificial Systems

To effectively integrate actomyosin into technological applications, external control is required to initiate, pause, or modulate its activity. In biological systems, this is handled naturally by signals that initiate and modulate cytoplasmic Ca^2+^ transients. In artificial environments, multiple strategies have been developed to achieve comparable precision in directing nanoscale movement.

#### 4.3.1. Biochemical Control (ATP and Calcium Ions)

This method replicates the native mechanism by using MgATP as an energy source and calcium to regulate myosin–actin interactions. In muscle and many non-muscle cells, calcium acts as a key trigger: when intracellular Ca^2+^ levels rise, the troponin–tropomyosin complex undergoes a conformational shift that exposes myosin-binding sites on actin, allowing contraction to proceed. Although Ca^2+^ is the physiological activator, other divalent cations such as Sr^2+^ and Ba^2+^ can partially substitute in activating thin filaments, typically with altered sensitivity and kinetics. These substitutions have been used experimentally to probe regulatory mechanisms [89,90,114]. In vivo, actomyosin regulation is further influenced by neurotransmitter signaling; for example, serotonin has been shown to modulate contractility and morphogenesis across divergent lineages [115].

In vitro assays that incorporate regulated actin filaments (actin decorated with tropomyosin and troponin) allow precise control of this interaction, making them ideal for studying how Ca^2+^ modulates thin filament activation. In most experimental systems, actin and myosin are purified from native sources, while regulatory proteins such as troponin and tropomyosin are more readily produced recombinantly and then combined with actin filaments to confer Ca^2+^-dependent regulation [92,93,99,101,102,116,117]. These systems have been widely used to assess both normal regulatory function and the impact of disease-associated mutations in Ca^2+^-sensitive proteins. For example, gliding filament assays can quantify changes in filament sliding velocity and activation thresholds, offering insight into how specific mutations alter contractile behavior [102,118,119,120,121,122,123,124,125].

Beyond Ca^2+^ regulation, nucleotide identity itself can modulate performance. Substitution of ATP with dATP, for instance, increases filament sliding speed and force generation, offering a simple yet powerful means of tuning actomyosin behavior in vitro. Such approaches may allow the design of nanosystems where motor output is optimized for specific applications [86,126,127]. New kinetic models have been proposed to reinterpret how actomyosin transduces energy under varying nucleotide and regulatory conditions, offering updated frameworks for designing such nanosystems [128,129,130].

Moreover, disease-associated mutations linked to hypertrophic cardiomyopathy (HCM)—such as those in cardiac troponin I or tropomyosin—can result in altered Ca^2+^ sensitivity, often shifting the activation curve and modifying the energy efficiency or kinetics of contraction [121,131]. These mutant proteins have been incorporated into nanomechanical assays primarily to model disease mechanisms and to reveal how pathological variants alter Ca^2+^-dependent regulation compared with healthy proteins; while such studies are currently research tools, they also suggest potential for future diagnostic platforms that differentiate physiological from pathological Ca^2+^ responses [101,117,132,133,134].

This approach offers high biocompatibility and reversibility, as movement ceases when calcium is removed [135,136]. However, response times are relatively slow, and broad calcium signaling may inadvertently affect other biological processes within hybrid systems.

Importantly, mutant-regulated filaments could also be integrated into biosensors or smart drug-delivery devices, where their unique calcium-response profiles might enable disease-specific actuation. For example, a device that differentially responds to local Ca^2+^ transients in heart failure versus healthy tissue could be designed using variants with altered Ca^2+^ activation thresholds.

#### 4.3.2. Thermal Control (Heat)

Localized heating—achieved using embedded microheaters or thermoresponsive surfaces—can trigger actomyosin activity within milliseconds [123,137,138,139]. Rapid activation and spatial targeting make this method attractive, especially for microdevice integration. However, assays must be conducted within a moderate thermal range: reliable Ca^2+^ regulation is observed between ~20–35 °C, while prolonged exposure above ~45 °C risks protein denaturation or damage to surrounding biomaterials [137].

Beyond mechanical activation, temperature can modulate the regulatory state of thin filaments, particularly those containing troponin and tropomyosin. Micropatterned heat pulses can trigger calcium-sensitive activation of cardiac thin filaments, with distinct roles played by each regulatory component [140,141]. In such assays, thin filaments are typically reconstituted from native actin and recombinant troponin/tropomyosin, allowing researchers to probe how temperature shifts modulate Ca^2+^-dependent regulation under controlled in vitro conditions [142,143]. These findings emphasize that the effect of heat is not limited to increased kinetic energy but includes specific molecular interactions between Ca^2+^-regulatory proteins.

Importantly, gliding filament assays using regulated actin have been used to study how temperature shifts modulate filament sliding. This approach enables comparison between healthy regulatory systems and variants linked to cardiomyopathy. For example, mutations in cardiac troponin T and I (e.g., R278C and R145G, respectively) can sensitize filaments to temperature changes, leading to aberrant activation even at physiologically mild heat elevations [121,131,137]. Such responses may have clinical relevance, as increased body temperature during exercise or fever could precipitate contractile dysregulation in individuals with these mutations.

This dual sensitivity to calcium and temperature provides opportunities for designing actomyosin-based devices that mimic physiological regulatory complexity—while also revealing risks for heat-induced dysfunction in disease models. Recent methodological advances have also extended the stability of actomyosin motility assays, enabling sustained function for many hours under optimized flow cell and buffer conditions [144]. Assays exploiting thermal gradients may thus serve both as diagnostic tools and as models for developing personalized therapies.

#### 4.3.3. Electrical or Electrochemical Stimulation

The application of electrical fields can modify pH (Stewart et al., 2021), ion concentrations [145], or initiate ATP synthesis, offering compatibility with silicon-based platforms. Such methods provide fast and programmable control, although unintended cross-talk with nearby components or pH-sensitive materials may limit selectivity [146].

#### 4.3.4. Light Activation (Optogenetics or Photothermal Methods)

Light-based systems offer non-invasive, reversible and highly localized control. For broader design principles and recent applications of photoactivated molecular motors, see Corra et al. [32]. Certain light-sensitive proteins undergo conformational changes in response to illumination, enabling control over actomyosin activity [147,148]. Photothermal nanoparticles offer an alternative route by converting light into heat, activating motors indirectly. Optogenetic tools have demonstrated precise activation of contraction in living tissue [149] and continue to show potential for expansion into fully artificial constructs [150]. In vitro, light activation is often implemented by integrating recombinant light-sensitive domains into regulatory proteins, which are then combined with native actin–myosin systems to create optically responsive constructs [151].

#### 4.3.5. Surface Patterning and Environment Control

Motion can be shaped by tailoring the substrate. Microchannels, chemical gradients and patterned coatings can confine and steer actin filaments along desired trajectories, functioning like nanoscale tracks. While this method does not initiate or halt motion directly, it provides spatial guidance and increases transport fidelity. Often demonstrated with kinesin and microtubules [152], similar techniques are applicable to actomyosin, too. Physical constraint and optimization strategies for patterning have been reviewed [12].

In most in vitro motility assays, myosin fragments—commonly heavy meromyosin (HMM) or subfragment-1 (S1)—are immobilized onto glass, plastic, or bead surfaces to serve as anchors for actin filament transport. Immobilization can be achieved through relatively simple approaches such as nitrocellulose coating, which promotes nonspecific adsorption while preserving activity [104]. More controlled chemistries include silanization of glass surfaces or the use of biotin–streptavidin linkages to couple biotinylated proteins with high specificity [153]. These strategies ensure stable attachment while maintaining enzymatic function. Site-specific immobilization using affinity tags has been developed to improve reproducibility and control of protein orientation. His-tagged myosin fragments can be linked via Ni-NTA chemistry and SNAP-tag–based strategies allow covalent coupling to functionalized substrates with defined geometry [154]. Such advances provide greater control over motor density, orientation and spatial localization, thereby extending the utility of actomyosin systems for biosensing and nanotechnological applications.

Each of these approaches has strengths and limitations. For instance, ATP/Ca^2+^ regulation mirrors natural control mechanisms but may lack the temporal precision of light-based triggers. Thermal and electrical systems offer speed and integration potential but may require careful calibration. Hybrid methods—such as combining surface patterning with photothermal activation—allow for increased control, enabling motion that is both guided and responsive. Confinement and surface topography can reorganize actomyosin networks, altering transport fidelity and collective behavior [155]. Table 3 summarizes the capabilities and trade-offs of these control modalities. Together, they provide the foundation for programmable actomyosin-driven nanodevices capable of precise, reversible and scalable operation within biomedical and engineering contexts.

### 4.4. Applications in Biomedicine and Engineering

The actomyosin system—where myosin motors move along actin filaments—acts like a tiny conveyor belt in eukaryotic cells. It helps to move materials around, to change cell shape, to guide movement and to build tissues [156]. This movement is powered by MgATP, the cell’s energy molecule, and is highly controlled and flexible [157,158,159,160].

Building on these fundamental properties and control mechanisms, recent work has begun applying actomyosin systems directly in sensing, diagnostic and therapeutic contexts. Their unique responsiveness to physiological signals—such as Ca^2+^, MgATP and temperature—makes them ideal candidates for developing bio-integrated platforms that can both detect and respond to changes in their environment. In what follows, we explore how these biological motors have been reconstituted into experimental systems and progressively adapted for real-world applications, including targeted delivery, responsive actuators, soft robotics, neural interfaces and biosensors.

Several natural processes show how this system works:

(1) During early development, cells use actomyosin to pinch and fold into complex tissue shapes [161].
(2) In cell division, it tightens like a drawstring to divide one cell into two [162].
(3) Large cells, like egg cells, use it to stir their contents [163].
(4) In the ear, actomyosin helps to maintain delicate structures that let us hear [164].

Inspired by these functions, actomyosin-based systems have been successfully reconstituted in laboratory environments (see Section 3.2). When actin filaments are introduced onto specially prepared surfaces containing immobilized myosin motors, they glide across the surface in a highly organized manner [49,50,165]. These systems form the basis of programmable molecular transport platforms, enabling precise control over nanoscale motion, and can be used for:

(1) Delivering medicine: Actin filaments can carry tiny drug packages to specific locations [11,12,166,167,168].
(2) Sorting materials: Lab-on-a-chip devices can direct different molecules or particles to the right spot [169].
(3) Detecting chemicals: The way actin moves can signal if certain biological substances are present [170].

These applications show how actomyosin can bridge the gap between biology and engineering—offering precise movement at the microscopic scale.

#### 4.4.1. Bioactuators and Contractile Elements

Actomyosin has been integrated into functional components of soft robotics and engineered tissues. For instance, actomyosin-generated force has been used to drive the mechanical displacement of microscale components [171]. Similarly, soft robotic elements derived from living muscle cells have been constructed to bend and even undergo partial self-repair [172].

Because actomyosin responds predictably to biochemical cues such as calcium ions and physical stimuli like temperature, it can be triggered with a high degree of control. Moreover, it operates through repeated cycles of contraction and relaxation with minimal loss of performance over time—a feature not always matched by synthetic analogs. In certain use cases, biologically derived actuators have been shown to outperform conventional mechanical alternatives, particularly in applications requiring flexibility or biocompatibility [173].

Among the biochemical cues, calcium plays a particularly powerful and tunable role. Reconstituted thin filaments containing troponin and tropomyosin exhibit clear, threshold-like responses to calcium, allowing engineered systems to mimic cardiac or skeletal muscle behavior. Gliding filament assays have been used not only to evaluate these dynamics but also to introduce disease-linked mutations into troponin components, generating constructs with unique calcium sensitivity [121,125,137]. These reconstituted systems typically pair native actin and myosin with recombinant troponin variants, allowing direct comparison between healthy and disease-linked regulation under identical assay conditions [101,117,132]. Such filaments can be used to develop actomyosin-based actuators that are selectively triggered by disease-relevant calcium ion concentration ranges.

#### 4.4.2. Electrically and Thermally Triggered Systems

Heat and electricity can also be used to control actomyosin motion. Tiny heaters have been used to turn movement on and off with precise timing, mimicking how muscles contract and relax. These systems responded to electrical current-induced changes in temperature that occur in less than a second [137,174]. This contrasts with or is possibly compatible with other strategies where chemical and physical cues control protein deposition and provide guidance to assembly and filament motion [168,175,176,177,178,179,180,181]. Such systems can be integrated into silicon chips—suggesting a path toward compact, programmable devices powered by biological motion [182].

Thermal control of actomyosin can be especially informative when regulated actin filaments are used, allowing the simultaneous interrogation of mechanical performance and biochemical responsiveness. For example, Ishiwata’s group has shown that heat can directly activate cardiac thin filaments in a Ca^2+^-dependent manner, and that both myosin and troponin-tropomyosin contribute distinctively to this thermal responsiveness [140,141]. Such systems are particularly well-suited for in vitro assays that explore subtle temperature-induced regulatory transitions.

In the context of inherited cardiac disorders, thermal assays offer a unique lens to observe how pathogenic variants alter the temperature–Ca^2+^ activation relationship. Variants associated with hypertrophic cardiomyopathy—such as those in α-tropomyosin [125] or troponin [121,137]—can cause thin filaments to activate abnormally at elevated temperatures, even without full calcium saturation. These effects may help to explain clinical symptoms triggered by exercise or fever, including arrhythmia or sudden cardiac events in mutation carriers.

By integrating these disease-linked regulatory proteins into thermally controlled platforms, engineers can create devices that mimic pathological conditions and serve as early detection or screening tools. In a broader sense, these findings also suggest a future in which actomyosin-powered actuators respond not only to intentional thermal input but also to physiological temperature fluctuations—making them relevant for patient-specific biosensors and biohybrid implants.

#### 4.4.3. Biosensing Applications

Actomyosin systems are very sensitive to their environment. Changes in acidity (pH), temperature, MgATP, or calcium levels can all influence how fast actin filaments move [92,137,183,184]. For example, sliding speeds change depending on how much MgATP is present [185,186]. Filaments begin to slide only after Ca^2+^ levels exceed a certain threshold, making actomyosin a potential biological sensor for detecting local biochemical states [187].

These finely tuned sensitivities allow actomyosin-based systems to operate not only as mechanical components but also as signal transducers. Practically, such biosensing assays are realized by immobilizing myosin fragments on patterned surfaces and introducing fluorescently labeled actin filaments with or without recombinant regulatory proteins, so that sliding velocities or activation thresholds can be measured as direct biosensor outputs [104,188]. Beyond velocity readouts, antibody-modulated actin motility has been used to develop proof-of-principle biosensors where binding events directly halt filament movement, offering a sensitive and label-free detection mode [189]. Their movement patterns can encode environmental information, forming the basis of biosensing functions that are directly coupled to mechanical readouts. This sensitivity has been leveraged in microfluidic platforms—miniaturized systems that manipulate fluids through narrow channels. These devices can detect target compounds in real time based on how they affect filament dynamics. One method uses carbon nanotubes to monitor actin–myosin interactions without the need for fluorescent tags or dyes [170].

Magnetically tagged biosensing strategies, such as those using quantum-well micro-Hall sensors to detect single magnetic beads, offer another label-free modality that is compatible with miniaturized actomyosin devices [190,191,192,193].

By embedding actomyosin elements into integrated platforms, it becomes possible to construct hybrid biosensors that convert biochemical input into visible, mechanical, or electrical output—broadening the toolkit for diagnostic, therapeutic and environmental sensing applications.

One especially important clinical application is in cardiovascular diagnostics. Cardiac troponins—particularly troponin I and T—are released into the bloodstream following myocardial injury and are the gold-standard biomarkers for diagnosing myocardial infarction [194,195,196,197]. Current diagnostic assays often rely on antibody-based detection. However, nanoscale and label-free platforms are emerging as faster, more sensitive alternatives. For example, functionalized SnO_2_ nanobelt field-effect transistor (FET) sensors have been developed to detect cardiac troponin without labels, using electrical readouts that respond to molecular binding [198].

Looking forward, actomyosin-based components could complement such platforms by serving as active sensing elements that respond mechanically to the presence or concentration of troponin or to shifts in calcium levels that often accompany cardiac stress. In principle, filaments reconstituted with disease-associated variants of troponin could be used to model pathological contractile responses—offering a dual readout of presence and functional consequence.

Thus, actomyosin-driven biosensors could be adapted not only for detecting circulating cardiac biomarkers but also for mimicking physiological or pathological mechanical responses in cardiovascular tissue. This dual capability makes them promising tools for real-time monitoring, early diagnosis and possibly even therapeutic feedback control in heart disease management.

#### 4.4.4. Vision and Neural Interfaces

Beyond mechanical functions, actomyosin contributes to key processes in the nervous system, including tissue repair and neurodevelopment. In the retina, it supports nerve regeneration; suppressing specific myosin isoforms has been shown to facilitate axon regrowth after injury [199]. Within the brain, actomyosin helps to sculpt synaptic connections and guide developing neurons as they form networks [186].

Tension generated by actomyosin has also been used to stretch synthetic membranes in a controlled way, suggesting new strategies for building soft, adaptive neural interface materials. Such systems have the potential to bridge engineered devices with fragile neural tissues—providing a foundation for future technologies such as visual prosthetics and brain–machine interfaces [200].

Together, these neural and sensory applications highlight how actomyosin systems can be harnessed not only for mechanical tasks but also for dynamic interaction with the nervous system. As research progresses, this opens the door to more speculative concepts—where actomyosin-based tools might one day interface with emotional or social cues, offering responsive, human-centered technologies. The next section explores such forward-looking possibilities.

#### 4.4.5. Looking Ahead: Emotion-Responsive Devices

One exciting and speculative direction is the development of emotion-sensitive systems using actomyosin. These envisioned devices could detect human emotional cues—such as facial expressions—and respond with controlled mechanical or chemical outputs. For example, if a face shows sadness or stress, a rise in Ca^2+^ or a bit of light could activate actomyosin in the device—leading to a gentle mechanical response, or even releasing helpful molecules. In practice, the actomyosin module would not “sense” emotion directly; rather, a front-end biosensor (e.g., camera plus expression-recognition software or a wearable detecting stress-linked biomarkers) would detect the cue and generate a digital control signal that then triggers the actomyosin actuator via light or local Ca^2+^ delivery. This might help people with social difficulties, like those with autism, by offering supportive feedback in real time [201,202,203]. We emphasize that such systems remain conceptual and would require integration of established sensing modalities with downstream actomyosin actuation.

Although no such devices currently exist, foundational components are rapidly advancing. Recent progress in optogenetically controlled actomyosin systems—such as OptoMYPT [148], a light-activated tool that modulates cellular contractility by targeting the myosin phosphatase pathway—alongside programmable bio-motors, provides a feasible technical basis for future emotion-aware biohybrid interfaces. In parallel, functionalized SnO_2_ nanobelt field-effect transistor (FET) sensors illustrate how hybrid devices can already achieve real-time biochemical monitoring. These FET sensors operate by transducing binding events of cardiac biomarkers (e.g., troponin I) at their functionalized surface into electrical conductance changes [204]. In practical use, they are typically exposed to small volumes of blood or serum samples rather than placed directly on cardiac tissue, though in vitro tissue studies have also applied the same principle under controlled conditions [205]. Such technologies demonstrate the potential of biohybrid systems to serve as diagnostic or monitoring tools in heart disease management.

While still in the conceptual stage, these developments suggest that actomyosin-powered technologies may ultimately extend beyond sensing and actuation—toward emotionally intelligent systems that interact dynamically with human users in both clinical and everyday contexts.

In summary, actomyosin-powered tools are moving from the lab bench into real-world applications—from biosensors to soft robots, from nerve repair to potential emotion interfaces. These biological motors, powered by nature and controlled by science, offer exciting possibilities for the future of medicine, engineering and human–technology interaction.

### 4.5. Engineering Challenges and Design Considerations

While actomyosin systems hold great promise, turning them into reliable tools for nanotechnology is not simple. Several key challenges need to be addressed for these biological machines to work effectively in artificial environments.

#### 4.5.1. Stability over Time

Proteins like actin and myosin are sensitive to environmental conditions. Factors such as heat, air exposure and shifts in chemical composition can degrade their function over time. In experimental setups, fluctuations in pH and/or inorganic phosphate (Pi) concentration [183,206,207,208,209,210,211,212,213,214,215] or oxygen levels [216] can disrupt activity and lead to system failure. To address this, several strategies have been developed—including the use of sealed enclosures, buffering agents and oxygen scavengers—to help preserve protein integrity over extended periods [170].

#### 4.5.2. Proper Attachment and Orientation

Effective operation of actomyosin systems depends on the spatial arrangement of myosin motors. When motors are unevenly distributed or randomly oriented on a surface, actin filament movement becomes erratic or stalls. To mitigate this, surface-engineering approaches have been developed to encourage uniform attachment and maintain consistent activity over time [217].

#### 4.5.3. Spatial Control

In biological systems, motor activity is often regulated locally through spatially confined signals. In contrast, in vitro systems tend to expose the entire surface to uniform inputs, such as ATP or calcium, which limits control over where and when movement occurs. This challenge can be addressed through the use of microscale patterning, chemical gradients, or physical channeling to guide actin filament trajectories along predefined paths—effectively creating tracks for molecular transport [175,218].

#### 4.5.4. Scaling up

When many motors work together, cooperative phenomena and new problems can emerge. Too many motors in one area might slow things down or cause unexpected traffic jams. This is why computer simulations are useful for testing designs before building them [219]. These models help predict how motor systems will behave when scaled up to more complex devices.

#### 4.5.5. The Path Forward

Overcoming these challenges will require combining several tools:

(1) Better chemistry to keep proteins stable;
(2) Advanced surfaces for guiding movement;
(3) Smarter control systems using light, heat, or electricity;
(4) Integration with readout mechanisms for biosensing applications;
(5) Computer modeling to plan system-wide behavior.

As efforts move from proof-of-principle devices to field-deployable tools, actomyosin systems may increasingly support multifunctional platforms where sensing, computation and actuation are performed by a single, biologically integrated module.

## 5. Conclusions

The actomyosin system—nature’s versatile molecular motor—is emerging as a powerful tool for the next generation of nanotechnology and biomedical devices. Its ability to generate force and movement with high efficiency, under biocompatible conditions, gives it a distinct advantage over many synthetic systems. From drug delivery and biosensing to soft robotics and potential neural interfaces, actomyosin-powered technologies are no longer confined to the lab—they are on the cusp of real-world applications.

However, turning biological motors into reliable tools for engineering remains a complex challenge. Issues of stability, scalability and precise control must be addressed through interdisciplinary innovation—combining protein chemistry, surface science, electrical and optical engineering, and computational modeling. Recent advances in optogenetics, electrochemical control and nanoscale fabrication show that these hurdles are not insurmountable [27].

In particular, the use of actomyosin as both a mechanical engine and a responsive sensor opens new possibilities for dynamic, feedback-driven devices in diagnostics and monitoring. The system’s innate sensitivity to activating Ca^2+^—as well as to other activating ions such as Sr^2+^ or Ba^2+^—substrate MgATP—and alternative hydrolysable nucleotide substrates such as dATP—and products MgADP, Pi and pH makes it a natural fit for biosensing platforms that require selective, real-time readouts in biological environments. Sensitivity to MgADP can be enhanced by using buffer systems such as that found in muscles and other cells, the Lohmann reaction in which the enzyme creatine kinase catalyzes the reversible transfer of Pi from phosphocreatine (PCr) to MgADP to form MgATP (and Cr), thereby lowering MgATP levels and increasing the free energy of MgATP hydrolysis, ΔG_ATP_ [210]. Similarly, sensitivity to Pi can be enhanced by using a buffer system for Pi (“phosphate mop”) such as generation of ribose-1-phosphate (and 7-methylguanine) from the substrate 7-methylguanosine in the presence of Pi by the enzyme purine nucleoside phosphorylase [220,221], which also increases the free energy of MgATP hydrolysis, ΔG_ATP_ [210].

**Figure 4:**
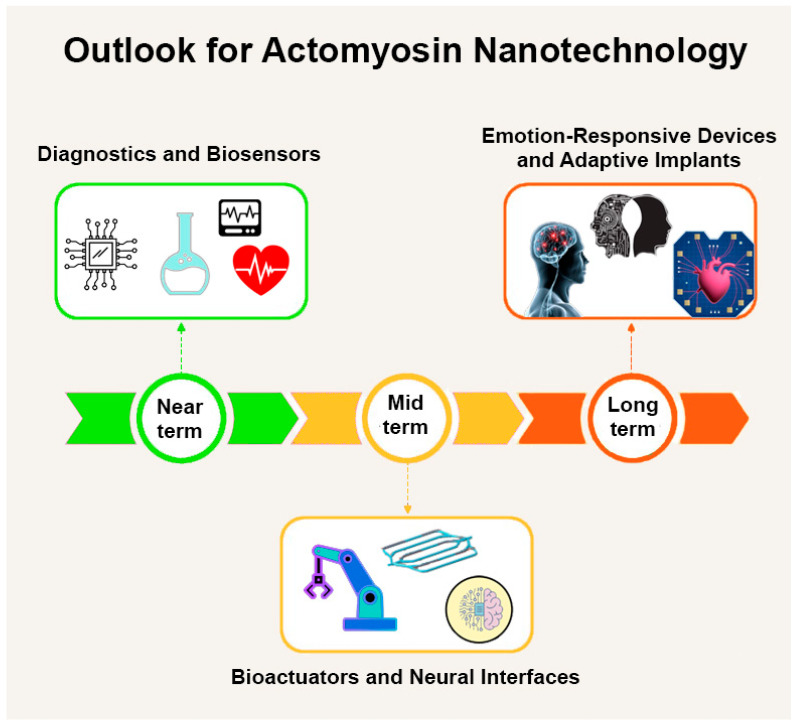
Roadmap for actomyosin-based nanotechnology. Near-term applications center on diagnostics and biosensing, leveraging actomyosin’s sensitivity to physiological signals. Mid-term opportunities include integration into bioactuators, soft robotics and neural interfaces, where contractile function and adaptability can be harnessed for engineered systems. Looking further ahead, speculative directions include emotion-responsive biohybrid devices, adaptive implants and human–machine symbiotic technologies. Together, these stages illustrate the trajectory from current laboratory prototypes to visionary biomedical applications.

As summarized in Figure 4, the development of actomyosin-powered nanodevices can be envisioned as a roadmap: from near-term applications in diagnostics and biosensing, through emerging integration into bioactuators and neural interfaces, to long-term speculative possibilities such as emotion-responsive devices and adaptive implants.

As researchers continue to refine these systems and build hybrid devices that merge the biological and artificial, actomyosin stands out not only for its technical potential but also for its conceptual elegance: a reminder that the solutions to tomorrow’s technological problems may already exist within our cells. The future of nanotechnology may well lie in understanding, adapting and amplifying what evolution has already perfected.

## Figures and Tables

**Table 1:** Nanomachine classes.

Class	Example(s)	Actuation Trigger	Potential Biomedical Applications
Natural Protein Motors	Myosin, Kinesin, Dynein	ATP, GTP	Targeted transport, neural interfaces, synthetic muscles
Rotary Enzymes	F1-ATPase, bacterial flagella	Proton gradient, ATP hydrolysis	Bioelectronic coupling, molecular stirring/mixing
DNA-Based Walkers	DNA walkers, spiders	Strand displacement, enzyme fuel	Molecular diagnostics, intracellular tracking
Synthetic Molecular Motors	Feringa motors, rotaxanes, catenanes	Light, redox, pH	Drug release, chemical computation
Stimuli-Responsive Polymers	PNIPAM, polyacrylamide derivatives	Heat, pH, light	Drug delivery, dynamic scaffolds
Nanoparticle Systems	Magnetic NPs, gold NPs, quantum dots	Magnetic field, light	Imaging, hyperthermia, theranostics

**Table 2:** Natural Molecular Motors.

Motor Protein	Track	Directionality *	Typical Step Size	Velocity	Primary Function	Nanotech Applications
Myosin	Actin filaments	+ end (varies by isoform); − end (e.g., Myosin VI)	5–36 nm	~0.1–5 µm/s	Muscle contraction, short-range intracellular transport	Bioactuators, contractile scaffolds, responsive biosensors
Kinesin	Microtubules	+ end (most isoforms); − end (e.g., Kinesin-14)	8 nm	~0.5–2 µm/s	Anterograde transport (e.g., vesicles, organelles)	Cargo transport on microtubule tracks, molecular sorters
Dynein	Microtubules	– end	8–32 nm	~1–14 µm/s	Retrograde transport, mitosis, ciliary and flagellar motion	Directional switches, retrograde cargo delivery, motile microsensors

* Directionality refers to the predominant behavior within each motor protein family. Notable exceptions include Myosin VI, which moves toward the minus (–) end of actin filaments, and members of the Kinesin-14 family (e.g., Kar3 in yeast, KIFC1 in mammals), which move toward the minus end of microtubules. To date, all known dyneins are minus-end directed.

**Table 3:** Summary of Control Modalities.

Control Method	Precision	Reversibility	Biocompatibility	Scalability	Notes
ATP/Ca^2+^ Regulation	Moderate	High	High	High	Direct physiological mimicry
Thermal Control	High	High	Moderate	Moderate	Strong for MEMS * integration
Electrical Stimulation	High	Moderate	Moderate	High	Interface-ready but risk of side effects
Optical/Photothermal	Very High	High	High (with care)	Moderate	Allows contact-free, localized control
Surface Patterning	Moderate	Low	High	High	Ideal for guidance, not activation

* MEMS = Microelectromechanical Systems: miniature devices that integrate mechanical and electrical components, often used in sensors, actuators, and lab-on-a-chip platforms.

## Data Availability

No new data were created or analyzed in this study.
